# A Review of Nitrates in Drinking Water: Maternal Exposure and Adverse Reproductive and Developmental Outcomes

**DOI:** 10.1289/ehp.8407

**Published:** 2005-11-03

**Authors:** Deana M. Manassaram, Lorraine C. Backer, Deborah M. Moll

**Affiliations:** Centers for Disease Control and Prevention, National Center for Environmental Health, Health Studies Branch, Atlanta, Georgia, USA

**Keywords:** developmental outcomes, drinking water, groundwater, maternal exposure, nitrate, nitrite, private wells, reproductive health

## Abstract

In this review we present an update on maternal exposure to nitrates in drinking water in relation to possible adverse reproductive and developmental effects, and also discuss nitrates in drinking water in the United States. The current standard for nitrates in drinking water is based on retrospective studies and approximates a level that protects infants from methemoglobinemia, but no safety factor is built into the standard. The current standard applies only to public water systems. Drinking water source was related to nitrate exposure (i.e., private systems water was more likely than community system water to have nitrate levels above the maximum contaminant limit). Animal studies have found adverse reproductive effects resulting from higher doses of nitrate or nitrite. The epidemiologic evidence of a direct exposure–response relationship between drinking water nitrate level and adverse reproductive effect is still not clear. However, some reports have suggested an association between exposure to nitrates in drinking water and spontaneous abortions, intrauterine growth restriction, and various birth defects. Uncertainties in epidemiologic studies include the lack of individual exposure assessment that would rule out confounding of the exposure with some other cause. Nitrates may be just one of the contaminants in drinking water contributing to adverse outcomes. We conclude that the current literature does not provide sufficient evidence of a causal relationship between exposure to nitrates in drinking water and adverse reproductive effects. Future studies incorporating individual exposure assessment about users of private wells—the population most at risk—should be considered.

The health implications of exposure to nitrates in drinking water were first reported in the scientific literature by Comly in 1945 after observing cyanosis in infants in Iowa, where well water was used in formula preparation (Comly 1987). Since then, most studies on the health effects of nitrates in drinking water have focused on infants because they are thought to be the most vulnerable to this exposure. More recent evaluations of the health implications of nitrates in drinking water have examined reproductive and developmental effects (Table 1) (Arbuckle et al. 1986, 1988; Dorsch et al. 1984; Fan and Steinberg 1996; Fan et al. 1987; Gelperin et al. 1975; Scragg et al. 1982; Super et al. 1981; Tabacova and Balabaeva 1993; Tabacova et al. 1997, 1998).

Data are insufficient on the incidence of methemoglobinemia among infants in the United States and on the effects that exposure to nitrate levels above the maximum contaminant limit (MCL) may have on other populations such as pregnant women. We do know that people in the United States who get their water from public water systems are occasionally exposed to biologically relevant doses of nitrate in their drinking water [U.S. Environmental Protection Agency (EPA) 1990]. In addition, a considerable number of people (~ 16% of the U.S. population) use private water systems that are usually located in areas considered more vulnerable to nitrate contamination (U.S. EPA 2002a).

In this review, we summarize the experimental and epidemiologic studies on nitrates in drinking water with reference to adverse reproductive and developmental effects. Previous reviews by Fan and colleagues (Fan and Steinberg 1996; Fan et al. 1987) focused on nitrates in drinking water, methemoglobinemia, and reproductive toxicity. In this review, we expand on these previous works, with a focus on maternal exposure and reproductive effects. Because a drinking water source may play a crucial role in exposure, we also present a discussion on sources and occurrence of nitrates in drinking water in the United States.

## Nitrate and Drinking Water

Nitrate occurs naturally in soil containing nitrogen-fixing bacteria, decaying plants, septic system effluent, and animal manure. Other sources of nitrate include nitrogenous fertilizers and airborne nitrogen compounds emitted by industry and automobiles [U.S. Geological Survey (USGS) 1996c]. Nitrate penetrates through soil and remains in groundwater for decades (Spalding and Exner 1993; USGS 1999). Groundwater is the source for > 50% of drinking water supplies, 96% of private water supplies, and an estimated 39% of public water supplies (USGS 1996a).

### Factors affecting nitrate in drinking water.

People who get water from shallow wells (< 100 feet below land surface) in areas with well-drained soils and high nitrogen inputs (e.g., close proximity to agricultural areas) have an increased risk of exposure to nitrate-rich groundwater. Agricultural activities are the largest nonpoint sources of nitrate contamination of groundwater because of greater use of nitrogen fertilizer on crops and the trend toward concentrated animal farming (Spalding and Exner 1993; USGS 1996b). Private wells are usually shallower and closer to sources of nitrate contamination, whereas public supply wells are usually in deeper groundwater aquifers where contamination is less likely (USGS 1996a).

### Drinking water regulations.

The U.S. EPA sets the standards for water quality regulations written in the Safe Drinking Water Act (SDWA) and its subsequent amendments (SDWA 1974). The SDWA specifies MCL for contaminants, defined as the concentrations above which adverse human health effects may occur (U.S. EPA 2002b). The enforceable MCL applies only to public drinking water systems and government or privately run companies supplying water to at least 25 people or with ≥ 15 service connections (U.S. EPA 2003).

Drinking water standards are usually set at a fraction of the no observed adverse effect levels (NOAELs) because potential health risks are often unknown or hard to predict. The standards are based on data from experimental animal studies and available human health reports. A margin of safety is usually built into the standard to account for issues such as extrapolating from animals to humans. This safety factor (orders of magnitude) is usually higher if potential health effects are more uncertain [Risk Assessment Information System (RAIS) 1995]. The MCL for nitrate in drinking water was set at 10 mg/L nitrate–nitrogen (NO_3_–N) or 45 mg/L nitrate (NO_3_^−^), on the basis of 214 methemoglobinemia cases reported to the American Public Health Association for which nitrate concentration data were available (Walton 1951). Because the current MCL was nominally based on human exposure data, no uncertainty or modifying factors were used, so there is no safety factor built into the MCL for nitrates in drinking water (Johnson and Kross 1990; Walton 1951).

The appropriateness of the current MCL is questioned (Avery 1999). Complicating matters is the existence of methemoglobinemia in infants without exposure to water that contains elevated nitrate levels or any apparent exogenous nitrate or nitrite exposure. These reports suggested that bacterial infection and subsequent overproduction of nitric oxide, or other substances in drinking water, such as copper, cause methemoglobinemia in infants (Avery 1999; Felsot 1998; Hanukoglu and Danon 1996; Hegesh and Shiloah 1982). However, case reports persist of methemoglobinemia in infants related to well water containing nitrate levels above the MCL (Knobeloch et al. 2000). Private water systems are not regulated, and methemoglobinemia is not a reportable disease, so there is insufficient data on whether exposure to nitrate levels above the MCL is a problem among users of such systems.

### Occurrence of nitrates in drinking water.

The National Water Quality Assessment (NAWQA) program of the USGS assessed water quality of aquifer systems that cover the water resources of > 60% of the population in the contiguous United States. On the basis of the NAWQA findings, approximately 15% of shallow groundwater sampled beneath agricultural and urban areas had nitrate levels above the MCL. In comparison, < 10% of samples taken from 100–200 feet deep exceeded the MCL, and no sample was found to exceed the MCL in groundwater that was > 200 feet below the surface (USGS 1999). Other reports using the NAWQA data showed nitrate levels > 3 mg/L (report assumed levels of ≥ 3 mg/L because of contamination) in 28% of samples taken from public and private wells. More private wells sampled (11%) exceeded the MCL than did public wells (2%) (Squillace et al. 2002).

The U.S. EPA National Pesticide Survey (U.S. EPA 1992), which sampled private wells in 38 states and public water systems in 50 states, found 1.2% of public water systems and 2.4% of private wells exceeded the MCL for nitrate (Spalding and Exner 1993). From this survey, the U.S. EPA estimated that > 4 million people, including some 66,000 infants < 1 year of age, could be served by systems that exceed the MCL for nitrate (U.S. EPA 1992). A survey by the Centers for Disease Control and Prevention (CDC) of > 5,500 private wells in nine midwestern states found nitrate levels above the MCL in 13.4% of wells sampled (CDC 1998). A survey of 3,351 domestic wells found that 9% had nitrate levels exceeding the MCL, compared with 1% of public wells (USGS 1995).

Although varied levels of nitrate in drinking water sources have been reported from state-based and national studies, a relationship between levels of nitrate and source of water is consistent. Specifically, higher levels of nitrate were found more often in ground-water than in surface water, in private wells than in public water supplies, in shallow wells than in deep wells, and in agricultural than in urban areas. The higher levels of nitrate in private water systems are of public health concern because users could be exposed to nitrate levels above the MCL.

## Materials and Methods

We conducted a Medline (PubMed 2005) search to identify publications in English through January 2004. Key words and phrases used to search the database for both animal and human studies included nitrates, nitrites, drinking water, pregnancy, fetus, birth defects, spontaneous abortions, prematurity, stillbirth, health effects, low birth weight, growth, reproductive outcomes, and drinking water contaminants. We also searched for internal publications from the CDC, USGS, and U.S. EPA via the agencies’ websites.

## Nitrate Toxicity

Nitrate toxicity is related primarily to the *in vivo* conversion to nitrite after ingestion [National Academy of Sciences (NAS) 1977; Swann 1975]. The health hazards from consuming water with nitrate are related to the direct toxicity of nitrite—that is, its ability to directly oxidize hemoglobin, changing it to methemoglobin, which cannot bind oxygen. Accumulation of methemoglobin (methemoglobinemia) occurs if this oxidation process overwhelms the protective reduction capacity of the cells (Jaffe 1981; Swann 1975). In addition to drinking water, which contributes an estimated < 3–21% of the average adult intake of nitrate, other sources include vegetables, meat, and meat products preserved with sodium nitrite [Agency for Toxic Substances and Disease Registry (ATSDR) 2001; Wogan et al. 1995]. There is also evidence of endogenous nitrate formation from the oxidation of nitric oxide independently of dietary sources (Walker 1996). A further concern relating to the metabolism of dietary nitrate is the potential *in vivo* formation of *N*-nitroso compounds from nitrite (Swann 1975). Nitrite is also a substrate in the formation of *N*-nitroso compounds from nitrosatable drugs. Therefore, higher dietary nitrate intake results in greater amounts being formed if there is concurrent exposure to nitrosatable drugs (Brender et al. 2004).

## Reproductive Toxicity

Animal studies have shown some indication that nitrate, nitrite, and *N*-nitroso compounds may traverse the placenta and affect the fetus *in utero* (Bruning-Fann and Kaneene 1993; Fan et al. 1987; Gruener et al. 1973). It has been suggested that the placental membrane is effective in separating blood circulation between mother and fetus from the fourth month of pregnancy, thus preventing methemoglobin molecules from crossing (L’hirondel and L’hirondel 2002). Others have suggested that nitrate or the reduced form (nitrite) may pass to the fetus through a system of active transport similar to that of iodide, and fetal nitrate plasma levels may exceed that of the mother (Hartman 1982). Tarburton and Metcalf (1985) found that amyl nitrite caused both adult and cord blood to oxidize from hemoglobin to methemoglobin *in vitro*. Cord blood was oxidized at a 5- to 6-fold greater rate than was adult blood. Whether nitrite may have the same effect *in vivo* and on fetal blood if it traverses the placenta is uncertain (Tarburton and Metcalf 1985).

### Animal studies.

Fan et al. (1987) reviewed the experimental data on reproductive toxicity and reported no evidence of teratogenic effects but found indication that nitrates and nitrites may induce abortion in experimental animals (Fan and Steinberg 1996; Fan et al. 1987; NAS 1981). These findings include increased levels of nitrite and methemoglobin in rats and subsequently in the fetuses when pregnant rats were given 2.5–50 mg/kg sodium nitrite in drinking water or through intraperitoneal injection. The possibility of increased permeability from placental damage was also tested in this study by giving another group of pregnant rats sodium nitrite after labor had begun. The first newborn had a normal methemoglobin level (1.2%), whereas those born after the chemical was given had higher levels of methemoglobin (10.1%) in their blood (Gruener et al. 1973).

Globus and Samuel (1978) orally administered 0.5 mg/day sodium nitrite to pregnant mice beginning the first day of gestation and continuing up to the 14th, 16th, or 18th day of gestation. The parameters used to measure embryotoxicity (litter size, gross anatomical defects, weight, number of resorption sites, proportion of fetal deaths) showed no significant difference between this group of mice and the control group given distilled water. The occurrence of skeletal abnormalities was similar in both groups; however, an increase in the production of red blood cells was reported for the offspring of treated mice. The investigators suggested that sodium nitrite may itself traverse the placenta, inducing methemoglobinemia in the fetus that could stimulate erythropoiesis in the hepatic cells (Globus and Samuel 1978).

Inui et al. (1979) administered doses of 125, 250, and 500 mg/kg sodium nitrite to hamsters on the 11th or 12th day of pregnancy. Other pregnant hamsters were given similar doses of sodium nitrate or similar doses of dimethylnitrosamine (positive controls). No chromosomal changes occurred in the offspring of animals treated with lower doses of sodium nitrite, but mutation occurred at the highest dose of sodium nitrite (500 mg/kg). The same dose of sodium nitrate did not produce such effects. Morphologic and neoplastic changes were observed in embryonic cells from hamsters given doses of 250 and 500 mg/kg sodium nitrite. The effects in pregnant hamsters treated with sodium nitrite were similar to those seen in the pregnant hamsters treated with dimethylnitrosamine as a positive control (Inui et al. 1979).

Cases of aborted fetuses were observed in pigs pastured in a feedlot of oats and rape. Serologic testing ruled out infectious causes for the abortions but found excessive nitrate. The report indicated that the levels of nitrate found in the rape (5.52%) and oats plants (0.53%) were not safe (Case 1957). After observing spontaneous abortions in cattle grazing in weedy pastures, Sund (1957) placed 12 pregnant heifers in pastures with soil known to have high nitrogen levels and high-nitrate weeds and eight pregnant heifers in pastures where prior treatment had killed high-nitrate weeds. Ten of the 12 heifers in the untreated pastures, compared with one of eight in the treated pastures, aborted their fetuses.

Blood levels taken at weekly intervals showed low, fluctuating levels of methemoglobin in all the heifers. The study suggested no association between abortion and levels of methemoglobin. However, the report noted that the degenerative changes observed in several organs of the aborted fetuses were indicative of tissue anoxia, symptomatic of methemoglobinemia. Sund concluded that the nitrates in weeds either caused or were related to the incidence of abortion in cattle grazing in pastures with soil high in available nitrogen (Sund 1957).

Winter and Hokanson (1964) indicated that spontaneous abortion may not be a significant effect of chronic nitrate exposure at levels sufficient to induce methemoglobinemia in cattle. Fifteen heifers were given sodium nitrate daily in their feedings from 2 months of pregnancy until they aborted or gave birth. The dosage was adjusted weekly to maintain methemoglobin levels at 20–30% of total hemoglobin. Two heifers aborted (one abortion was caused by vibriosis), and two heifers died from acute nitrate poisoning.

Other adverse reproductive effects, such as mummified fetuses; lesions on the cervix, uterus, and placenta; and maternal death were also reported by earlier experimental studies reviewed by Fan et al. (1987). A more recent study examined the effects on embryo growth and viability in 48 cows between 2 and 8 weeks of gestation that were feeding on heavily fertilized grass containing high levels of nitrogen compared with those in control pasture (Laven et al. 2002). No evidence was found that embryo survival or growth was affected from 20 days onward in pregnant cows grazing in either pasture.

Two groups of cows, pregnant and non-pregnant, were fed either nitrate rations < 400 ppm nitrate in hay for 5 weeks or a ration of 4,000 ppm nitrate in hay for 8 weeks (Page et al. 1990). Serum progesterone levels were depressed in cows fed the higher nitrate rations. The depressed levels were more evident in nonpregnant cows, less in cows in early pregnancy, and absent in cows in mid-pregnancy. The investigators concluded that early reproductive toxicity from chronic nitrate exposure may be caused by depression of serum progesterone, but further studies need to be done to confirm this conclusion.

## Epidemiologic Studies of Reproductive Effects of Nitrate in Drinking Water

### Birth defects.

The effects of exposure to nitrates in drinking water on the incidence of birth defects have been evaluated in several epidemiologic studies (Table 1) (Arbuckle et al. 1988; Cedergren et al. 2002; Croen et al. 2001; Fan and Steinberg 1996; Fan et al. 1987). However, the results from epidemiologic studies addressing this topic are equivocal.

In a case–control study of Mexican-American women, Brender et al. (2004) examined nitrosatable drug exposure and the occurrence of neural tube defects (NTDs) in relation to dietary nitrites and nitrates. They examined 184 cases of NTD-affected pregnancies from the Texas Neural Tube Defects Projects and 225 women with normal live births. All participants were interviewed to obtain detailed dietary information, pericon-ceptional medication use, and drinking water source. The water sources of 110 women (43 cases and 67 controls) were also tested for nitrate. Nitrosatable drug use was reported as a risk factor for having an NTD-affected pregnancy [odds ratio (OR) = 2.7; 95% confidence interval (CI), 1.4–5.3]. Among those who had their water tested for nitrate, drinking water nitrate level ≥ 3.5 mg/L nitrate–N was associated with having an NTD-affected pregnancy (OR = 1.9; 95% CI, 0.8–4.9). The risk estimate increased drastically (OR = 14; 95% CI, 1.7–660) for women who took nitrosatable drugs and had nitrate levels ≥ 3.5 mg/L nitrate–N in their drinking water source. The authors concluded that because the level of nitrate in the water sampled was relatively low, and women were not asked about frequency and amount of water consumed, the amount of nitrate in the water directly contributed to the increased risk observed among women who used nitrosatable drugs (Brender et al. 2004).

A study of 71,978 infants born from 1982 through 1996 was conducted in a Swedish county served by 80 municipal water systems (Cedergren et al. 2002). The study assessed the possible association between mothers’ preconception or early pregnancy exposure to chlorination by-products and nitrate in public drinking water and incidence of congenital cardiac defects. The study population was identified through the Swedish Birth Registry and was limited to infants whose mothers used the municipal water system and had addresses for the preconception or early pregnancy period that could be geocoded. The Registry of Congenital Malformations provided information on cardiac defects. Additional data on the pregnancy, delivery, and newborn health were obtained from medical records and the hospital discharge registry. Exposure assessment was ascertained by using a geographic information system to link the study subjects to specific water supplies. Groundwater as a source of drinking water was reported as a potential risk factor for cardiac defects (adjusted OR = 1.31; 95% CI, 1.09–1.57). A very small and not statistically significant excess risk for cardiac defects was found to be associated with levels ≥ 2 mg/L nitrate–N compared with those < 2 mg/L nitrate–N (adjusted OR = 1.18; 95% CI, 0.97–1.44) (Cedergren et al. 2002).

A case–control study in California investigated the potential association between maternal exposure to nitrates in drinking water and diet before pregnancy and the risk of NTDs in the mothers’ infants (Croen et al. 2001). Case infants (538) with NTDs (both live and stillborn singleton births) born from 1989 through 1991 were selected from California’s birth defects program. Control infants (539) were live births with no malformations selected from each area birth hospital for the same time period. Exposure assessment was done through interviews with the mothers, which included a detailed beverage and dietary questionnaire, and the state’s community water systems data for sources serving their preconception addresses. The authors found an increased risk for NTDs among babies born to mothers living in areas where the drinking water nitrate level was above the MCL compared with those in areas below the MCL (OR = 2.7; 95% CI, 0.76–9.3). This association did not change after adjusting for other dietary nitrate intake (OR = 1.9; 95% CI, 0.73–4.7). Increased risk with increasing levels of nitrates was observed. Risk estimates were higher among groundwater users; however, other risk factors (e.g., Hispanic ethnicity, young age, low socioeconomic status, and no vitamin use) for NTDs were also more common among groundwater users.

In this study, the authors examined risk separately for anencephaly and spina bifida (Croen et al. 2001). Increased risk for anencephaly in babies was associated with their mother living in an area where the nitrate in drinking water was above the MCL (OR = 4.0; 95% CI, 1.0–15.4). This association was not substantially altered when adjusted for dietary intake of nitrate. A doubling in risk for anencephaly in babies whose mothers lived in areas where the nitrate level in groundwater was ≥ 5 mg/L compared with < 5 mg/L was observed. No increased risk was observed in users of mixed water (i.e., a combination of surface water and groundwater). There was no increased risk for spina bifida with any level of nitrate exposure or source of drinking water (Croen et al. 2001).

Fan and Steinberg (1996) summarized the studies conducted on maternal exposure to nitrates in drinking water and birth defects by Scragg et al. (1982) and Dorsch et al. (1984) in South Australia, Arbuckle et al. (1988) in New Brunswick, Canada, and Bove et al. (1992) in New Jersey. The study by Dorsch et al. (1984) suggested an increased risk of bearing a child with a congenital malformation among women whose homes were served by water with a nitrate concentration > 5 ppm. Although the effects of nitrate cannot be discounted, the finding that the risks associated with multiple defects were increased suggests possible multiple risk factors. The study by Arbuckle et al. (1988) reported a protective relationship for users of public and spring water sources compared with private sources, but the ORs were not statistically significant. Five water samples exceeded the Canadian MCL (44 ppm) for nitrate. Bove et al. (1992) reported a positive association with water contamination and NTDs but cautioned that the study did not provide sufficient evidence of causation for any of the contaminants in question.

These studies assessed exposure to nitrates in drinking water on the basis of the source of the water. The lack of individual exposure assessment—whether the women actually drank the water—is a limitation in some investigations. The presence of other substances, such as pesticides, toxic metals, and chemicals (e.g., chlorinated solvents and chlorinated disinfection by-products), in private and public water systems may be correlated with the presence of nitrate. Some of these constituents are reported as risk factors for congenital malformations (Bove et al. 1992, 2002).

### Spontaneous abortions.

Aschengrau et al. (1989) investigated the quality of community drinking water and the occurrence of spontaneous abortions among a group of women in the Boston, Massachusetts, area. The population, selected from 1976 through 1978, consisted of women who lived in Massachusetts during their pregnancy, lived in a town with a public water supply, and were admitted to a specific community hospital. Cases were 286 women who had a miscarriage during their first 27 weeks of pregnancy, and controls were 1,391 women who had live births. The women were interviewed to obtain demographic and behavioral information, and water quality data were obtained from public records of routine analyses of public tap water. All levels of nitrate were below the MCL. A negative association was reported for any detectable level of nitrate and the occurrence of spontaneous abortions. The report noted that risk estimates may have been diluted by the measurement, recording, and classification of exposure, because this information was obtained indirectly from public water supply records of the communities where the women lived at the time the spontaneous abortions occurred (Aschengrau et al. 1989).

Gelperin et al. (1975) evaluated data from 1959 through 1966 on infant and fetal deaths in 16 Illinois communities, nine of which had nitrate levels ranging from 43 ppm to 123 ppm (NO_3_) in their water supply. Communities were grouped into three categories, consistently having high nitrate (above the MCL), having high nitrate in spring only, or not having high nitrate levels. The community water supply data provided nitrate levels. No significant increase in fetal deaths was found in areas consistently reporting nitrate levels above the MCL in their water compared with other areas.

Skrivan (1971) measured methemoglobin levels in the blood of pregnant women over a 2-year period to evaluate whether the observed mean values of methemoglobin would be similar in women who experienced spontaneous abortions and in those who experienced term delivery. The mean values in women before spontaneous abortion did not differ significantly from the mean values among women with term deliveries (Skrivan 1971).

An earlier evaluation of methemoglobin levels in pregnancy reported a relationship between methemoglobinemia and miscarriages in humans (Schmitz 1961). The study tested methemoglobin levels in 25 women in their first trimester of pregnancy. Higher levels were observed in women who spontaneously aborted or who threatened abortion in the first trimester. The report noted nitrates and nitrites as the most common methemoglobin inducers and concluded that high maternal methemoglobin levels are possibly related to miscarriages.

### Case reports.

A report on a cluster of spontaneous abortions in LaGrange, Indiana, cited nitrate-contaminated water from private wells as the possible cause (CDC 1996). The cases included a 35-year-old woman who experienced four consecutive miscarriages and a 37-year-old and a 20-year-old who each experienced one miscarriage. All three women lived within 1 mile of each other and were in the first trimester of pregnancy at the time of the miscarriages. Testing of the wells serving the homes of the women found nitrate to be the only elevated contaminant. The wells had nitrate levels over the MCL, with reported levels of 19.0 mg/L, 26 mg/L, and 19.2 mg/L nitrate–N for the three women, respectively. Although these incidents of spontaneous abortion may have been related to the ingestion of nitrate contaminated drinking water, other possible explanations such as genetic defects in the fetuses and cluster by chance could not be ruled out (CDC 1996).

### Other reproductive effects.

Besides birth defects and prenatal mortality, reproductive toxicity includes less readily observed effects that may be influenced by chronic low-level exposure to a toxic substance. These effects include sterility, intrauterine growth restriction, premature birth, and complications of pregnancy. Several of these outcomes have been addressed in epidemiologic studies of the potential effects of nitrate exposure on reproductive health (Table 1). Hypothetically, the oxidation of hemoglobin to methemoglobin, which limits the oxygen-carrying capacity of the blood, may interfere with the course and outcome of pregnancy (NAS 1981; Tabacova and Balabaeva 1993; Tabacova et al. 1997, 1998).

Bukowski et al. (2001) conducted a population-based case–control study on singleton births that occurred from 1991 through 1994 to mothers who resided in Prince Edward Island, Canada, and who used municipal and private water systems. The study examined the potential impact of groundwater nitrate exposure on prematurity and intrauterine growth restriction (IUGR). The study included 210 cases of IUGR, 336 cases of premature births, and 4,098 controls that were identified through a Reproductive Care Program (RCP) database. The authors developed a nitrate level exposure map using data on public and private wells collected from 1990 through 1993. Premature birth was defined as birth at < 37 weeks of gestation, IUGR was defined as birth weight < 2,500 g at termed birth, and controls were defined as “normal” live births with no other diagnoses. The nitrate data were subdivided into six exposure categories on the basis of increasing nitrate–N levels, with the highest exposure having levels ≥ 5 mg/L. The study subject was assigned to an exposure group on the basis of the mother’s residential address at the time of delivery. The RCP database provided additional data including demographics, lifestyle, health, and reproductive history (Bukowski et al. 2001).

Bukowski et al. (2001) found a significant relationship between IUGR and higher nitrate levels. Using the lowest exposure category (median level of ≤ 1.3 mg/L nitrate–N) as the comparison group, an adjusted OR showed an excess risk for higher exposure categories with median levels 3.1 mg/L (OR = 2.31; 95% CI, 1.47–3.64) and 4.3 mg/L (OR = 2.56; 95% CI, 1.44–4.45). An increased but not significant risk for the highest exposure group of 5.5 mg/L (OR = 1.34; 95% CI, 0.31–3.99) was observed, as well as a significant dose–response association between nitrate exposure and prematurity. The authors reported excess risk estimates for the exposure categories with median levels of 3.1 mg/L (OR = 1.82; 95% CI, 1.23–2.69), 4.3 mg/L (OR = 2.33; 95% CI, 1.46–3.68), and 5.5 mg/L (OR = 2.37; 95% CI, 1.07–4.80). The dose–response relationship is fairly consistent and suggestive; however, assessing exposure to nitrate based on ecologic classification makes it difficult to interpret the findings exclusively in terms of water nitrate exposure (Bukowski et al. 2001).

In a study in Bulgaria, Tabacova et al. (1997) investigated the association between maternal exposure to environmental nitrogen sources and subsequent complications of pregnancy. The study was done in an area known to have nitrate levels in drinking water ranging from 8 to 54 mg/L nitrate–N (depending on the season and the water supply source), as well as in the food supply and oxides of nitrogen in ambient air. The study, which included pregnant women at ≥ 24 weeks’ gestation, analyzed blood methemoglobin levels as markers of exposure to nitrogen compounds.

Personal interviews with the women furnished medical and lifestyle histories. Only 16% of the study group experienced a normal pregnancy. The others experienced complications, including anemia (67%), threatened spontaneous abortion/premature labor (33%), and toxemia (23%), with some women having more than one of these diagnoses. Among women with pregnancy pathology, methemoglobin levels were 0.1–11.2% of total hemoglobin, compared with 0.4–2.8% in women with normal pregnancy. The mean methemoglobin levels for all categories of pregnancy complications were above the physiologic normal level of 2%, with the highest mean level occurring in the toxemia group. The report suggests an increased risk for pregnancy complications from exposure to nitrogen compounds. However, the lack of individual exposure assessment and the presence of several potential sources of exposure prevent implicating drinking water as a single source (Tabacova et al. 1997).

Tabacova et al. (1998) also evaluated mother–infant pairs in an area of Bulgaria with chronic exposure to pollution by nitrogen compounds, including drinking water nitrate levels of 8–54 mg/L nitrate–N in municipal water system, 13–400 mg/L nitrate–N in wells, and nitrogen oxides in ambient air. They examined methemoglobin levels as markers for exposure to nitrogen compounds, measures of oxidative stress, and the health status of infants at birth. Fifty-one mother–infant pairs were recruited from a local hospital; interviews were conducted with the mothers, and clinical records were reviewed for information on demographics, lifestyle, and medical factors. Maternal and cord blood were tested for methemoglobin and markers of oxidative stress. More than half of maternal blood and almost half of cord blood had methemoglobin levels > 2%. The study indicated a strong association between maternal methemoglobin and cord blood methemoglobin. Maternal and cord blood methemoglobin levels were higher in cases of abnormal birth outcomes (preterm birth, fetal distress, and low birth weight) than in cases of normal birth outcomes. The authors also reported an association between methemoglobin levels in cord blood and adverse birth outcome. Overall, the results show that nitrate exposure may have a role in adverse reproductive effects. However, the small sample size, multiple sources of exposure, and the lack of individual exposure assessment make the findings difficult to interpret in terms of nitrate exposure from drinking water per se. Other pollutants potentially associated with abnormal birth outcomes were not discussed (Tabacova et al. 1998).

Fan et al. (1987; Fan and Steinberg 1996) discussed the study by Super et al. (1981) that evaluated infant morbidity in a rural area of southwest Africa known to have wells with high levels of nitrates. Super et al. (1981) found no association between the incidence of premature birth or the size of infant at birth and living in an area with nitrate levels > 20 mg/L nitrate–N compared with areas with levels ≤ 20 mg/L. No association was found between birth weight, current or previous premature births, stillbirths, and spontaneous abortions and nitrate levels > 20 mg/L nitrate–N. However, increased deaths among infants previously born to mothers in regions with nitrate levels > 20 mg/L nitrate–N were reported.

## Summary and Discussion

The contamination of groundwater by nitrates is ubiquitous, but the magnitude of the risk posed to human health is still unclear. That the potential toxicity of nitrate in drinking water is largely based on studies using high concentrations of nitrite is a complication. Another complication is that human studies have provided little evidence that adverse health effects result from chronic exposure to low levels of nitrate in drinking water. Although the current MCL for nitrates is also in debate, the public drinking water supply rarely exceeds this limit. The lack of data on unregulated systems, however, is an important issue. The available data on the occurrences of nitrates in drinking water indicate that users of private water systems are most at risk for exposure to nitrate levels above the MCL. However, a lack of studies focusing on users of private water systems means that the extent of the problem is unknown.

Experimental animal studies on nitrate or nitrite (in the form of sodium or potassium nitrate/nitrite) and adverse reproductive and developmental outcomes provide moderate evidence for an association between exposure to nitrate and fetal loss, neonatal mortality, maternal toxicity, and decrease in number of litters and live births (Table 2). Epidemiologic evidence for increased risk for adverse reproductive and developmental outcomes in humans from exposure to nitrate in drinking water is sparse and suggestive at best. Nevertheless, the findings of excess birth defects in some studies (Table 1) suggest the need for further studies.

In most studies presented in this review, exposure to nitrates in drinking water was assessed primarily through water quality data for water systems serving women’s addresses during pregnancy or at time of delivery. Although this form of exposure assessment provides information in a timely and cost-efficient way, community-based water quality data provide only a rough estimate of individual exposure. This does not account for other issues in exposure assessment such as other sources of nitrate exposure, individuals not drinking tap water, or use of private water systems. The recent study by Brender et al. (2004) is an example of the extent of exposure assessment that should be considered when evaluating nitrate exposure and reproductive effects.

Drinking water contaminants other than nitrates have been reported to be associated with increased risk of adverse pregnancy outcomes (Bove et al. 1992, 2002). Identifying which contaminant in a community water system is associated with a particular adverse reproductive outcome when multiple contaminants are present is difficult. Future studies should conduct individual exposure assessments such as maternal interviews concerning water consumption habits in and outside the home and other risk factors such as occupational exposures or smoking.

Many studies on birth defects are also limited by the time between the end of pregnancy and the maternal interview. This is difficult for individual studies to overcome because birth defects are rare outcomes and prospective cohort studies may not be feasible to conduct. Prospective cohort studies on end points such as spontaneous abortions are more feasible and would provide knowledge about the potential effects of nitrates in drinking water on this outcome. Spontaneous abortion may be a more sensitive indicator of adverse reproductive effects from relatively low levels of drinking water contamination.

States with large numbers of private wells where groundwater is vulnerable to contamination should be encouraged to increase monitoring or surveillance of such systems. Future research could include long-term monitoring or surveillance of water systems vulnerable to contamination. This could provide valuable exposure assessment information to conduct studies on drinking water contaminants such as nitrates. A discussion on the appropriateness of the current MCL is beyond the scope of this review. However, future studies with improved exposure assessment (including other dietary sources and medications), adequate sample size, and evaluating endogenous nitrate exposure could help to determine whether nitrates in drinking water at the current MCL increase the risk for reproductive and developmental effects.

## Figures and Tables

**Table 1:** Summary of epidemiologic studies that evaluated exposure to nitrate in drinking water and reproductive and developmental effects.

Reference	No. of subjects	Exposure	Outcome	Results
Brender et al. 2004	184 cases, 225 controls	Drinking water nitrate level, dietary nitrates and nitrites, nitrosatable drugs	NTDs	Increased risk for NTDs shown with nitrosatable drug use (OR = 2.7; 95% CI, 1.4–5.3), and nitrate level in water ≥ 3.5 mg/L (OR = 1.9; 95% CI, 0.8–4.6)
Cedergren et al. 2002	71,978 infants	Drinking water source; address close to conception or early pregnancy	Congenital cardiac defects	OR = 1.18 (95% CI, 0.97–1.44); no excess risk for cardiac defects shown with an increase in nitrate levels
Bukowski et al. 2001	546 infant cases, 4,098 infant controls	Drinking water source, mother’s address at delivery	Premature birth, low birth weight, IUGR	Higher nitrate concentrations associated with prematurity (OR = 2.37; 95% CI, 1.07–4.80), and IUGR (OR = 2.56; 95% CI, 1.44–4.45)
Croen et al. 2001	538 infant cases, 539 infant controls	Mother’s address before and during first trimester, drinking water source, interview data on water consumption	NTDs	Moderate (OR = 1.9; 95% CI, 0.73–4.7) but not significant increased risk of NTDs with nitrate levels above the MCL, significant increased risk (OR = 4.0; 95% CI, 1.0–15.4) of anencephaly associated with nitrate levels above the MCL
Tabacova et al. 1997	61 pregnancies	Public drinking water source, NO_2_ in ambient air; based on address during pregnancy	Pregnancy complications	Methemoglobin levels significantly higher in women with anemia, toxemia, and threatened abortion/premature delivery (mean range 2.8–6.6%), compared with normal pregnancy (mean 1.3%)
Tabacova et al. 1998	51 mother–infant pairs	Public drinking water source, NO_2_ in ambient air, nitrate in food; based on address at delivery	Neonatal health status	Maternal and cord blood methemoglobin levels higher in cases of abnormal birth outcomes (preterm births, low birth weight). Mother’s methemoglobin was associated with cord blood methemoglobin (*r* = 0.79, *p* < 0.0001)
Arbuckle et al. 1988	130 infant cases, 260 infant controls	Household water sample, drinking water source	CNS defects	Higher nitrate levels in private well sources. Increased risk of having an infant with a CNS defect associated with exposure to nitrate exposure from private well sources (ROR = 2.30; 95% CI, 0.73–7.29)
Aschengrau et al. 1989	286 cases, 1,391 controls	Public drinking water source, address at pregnancy outcome	Spontaneous abortion	Any detectable level nitrates was associated with decrease in frequency of spontaneous abortion (OR = 0.5; 95% CI, 0.2–0.9).
Dorsch et al. 1984	218 infant case–control pairs	Address at delivery, water sample from address	Congenital malformations	The higher the level of nitrate in the water the mother consumed, the greater the risk estimate for having a child with a malformation. Women who consume mainly groundwater had a greater risk of having a child with a malformation (relative risk = 2.8; 95% CI, 1.6–4.4)
Scragg et al. 1982	699 perinatal deaths	Drinking water source	Deaths due to congenital malformations	Highest death rate in area served by drinking water source with higher nitrate levels
Super et al. 1981	486 infants	Drinking water well, interview data on water consumption	Premature birth, size at birth	No significant association between higher nitrate levels in well water and incidence of premature birth or size at birth
Gelperin et al. 1975	30,980 infants	Community drinking water source	Infant and fetal mortality	No increase in fetal or infant mortality in areas where community water contained excess nitrates

Abbreviations: CI, confidence interval; CNS, central nervous system; IUGR, intrauterine growth restriction; MCL, maximum contaminant level; NTD, neural tube defect; OR, odds ratio; ROR, risk odds ratio.

**Table 2:** Reproductive and developmental effects in animal studies that evaluated exposure to nitrate or nitrite.

Reference	Animal species	Exposure	Results summary
Laven et al. 2002	Cattle	High nitrate (as available nitrogen) in pastures	Grazing in high nitrate pasture did not affect embryo survival or growth Higher milk and plasma urea levels in cows grazing in high nitrate pastures
Bruning-Fann et al. 1996	Swine	Nitrate in drinking water	Nitrate detected in 53% of sampled wells, median concentration of 2.1 ppm No association between the nitrate level in drinking water and litter size, number of stillbirths, deaths, or other known illnesses
U.S. FDA 1972a, 1972b	Mice, rats, hamsters, rabbits	KNO_3_ or KNO_2_, NaNO_3_ or NaNO_2_ oral intubation	No effect on maternal or fetal survival, no incidence of birth defects
Sinha et al. 1971	Guinea Pigs	NaNO_2_ subcutaneous injection	Abortion induced at 60 mg/kg NaNO_2_ Maternal death at 70 mg/kg NaNO_2_ High methemoglobin levels in mothers and fetuses
Sleight 1967	Guinea Pigs	KNO_3_ or KNO_2_ in drinking water	Decrease in litter size and number of live births, and fetal loss observed in those given KNO_3_ No live births and aborted fetuses observed in those given KNO_2_
Winter 1964	Cattle	NaNO_3_, NaNO_2_, or hydroxylamine in feeding	Two of the 15 heifers fed NaNO_3_ died from acute nitrate poisoning, two aborted (one due to vibriosis)
Simon et al. 1959	Cattle	KNO_3_ or NaNO_2_ capsules	No abortions in heifers given small doses of KNO_3_ or NaNO_2_ Three heifers died and three aborted after given higher doses of KNO_3_
Case 1957	Pigs	Grazing in pasture on oats and rape high in nitrate	Spontaneous abortion and stillbirths in offspring Negative test for infectious disease but high levels of nitrate found in the mothers’ blood
Sund et al. 1957	Cattle	Grazing in pastures where soil had high levels of available nitrogen, and weeds had high nitrate levels	10 of 12 heifers in weedy pastures aborted No association between methemoglobin levels and abortion

Abbreviations: KNO_2_, potassium nitrite; KNO_3_, potassium nitrate; NaNO_2_, sodium nitrite; NaNO_3_, sodium nitrate.
